# Post-Stroke Olfactory Dysfunction: Network Mechanisms, Clinical Consequences, and Rehabilitation

**DOI:** 10.3390/brainsci16101092

**Published:** 2026-10-10

**Authors:** Thomas Heinbockel, Gursimran Virdi, Lexington Shannon, Sayan Nandi

**Affiliations:** Department of Anatomy, Howard University College of Medicine, Washington, DC 20059, USA; theinbockel@howard.edu (T.H.); virdigursimran07@gmail.com (G.V.); lexington.shannon@bison.howard.edu (L.S.)

**Keywords:** anosmia, dysosmia, flavor, hyposmia, insula, olfaction, olfactory bulb, piriform cortex, olfactory training, orbitofrontal cortex, rehabilitation, smell dysfunction, thalamus

## Abstract

**Highlights:**

**What are the main findings?**

- Even though our sense of smell is critical for behavior and as a marker in neurological disease, it is not often evaluated in stroke patients.
- Due to its broad anatomical organization, post-stroke olfactory deficits may result from direct or indirect injury to olfactory and other brain structures.
- Olfactory dysfunction as a result of stroke may present as lasting impairment that persists into the chronic phase of stroke.

**What are the implications of the main findings?**

- Post-stroke olfactory dysfunction should be considered a multidimensional network-based disorder rather than a simple binary loss of smell.
- Recovery of smell function should be considered in rehabilitation efforts after stroke.

**Abstract:**

Stroke is the second leading cause of death after ischemic heart disease, accounting for 10% of all deaths worldwide. Stroke is also a major cause of long-term neurological disability, yet olfactory dysfunction remains an underrecognized consequence of cerebrovascular injury. Smell is rarely evaluated in stroke patients despite its central role in appetite, hedonia, hazard detection, memory, emotional processing, and quality of life. The olfactory system extends from the olfactory epithelium and olfactory bulb to primary olfactory cortex, limbic regions, orbitofrontal cortex, insula, thalamus, hippocampus, broader fronto-limbic networks and cerebellum. The broad anatomical organization of the olfactory system subjects it to frequent perturbation due to stroke or other lesions. Post-stroke olfactory deficits may result from direct injury to olfactory structures, disruption of higher-order olfactory networks, diaschisis, altered sniffing and sensorimotor control, or secondary degeneration. Clinically, these deficits are manifested as hyposmia, anosmia, impaired odor identification, discrimination and memory, altered odor pleasantness, reduced flavor perception, loss of appetite, or reduced odor-guided behavior. Current evidence indicates that olfactory dysfunction can persist into the chronic phase of stroke, although a single prevalence estimate cannot be established because populations, timing, testing methods, and reporting differ substantially across studies. This narrative review provides an overview of stroke-related olfactory dysfunction, emphasizing olfactory pathway anatomy, lesion-relevant mechanisms, clinical consequences, measurement limitations, rehabilitation approaches, and future research priorities. We argue that post-stroke olfactory dysfunction should be understood as a multidimensional network-based disorder rather than a simple binary loss of smell.

## 1. Introduction

Stroke is an acute neurological injury caused by interruption of cerebral blood flow or bleeding into brain tissue. It remains one of the leading causes of death and long-term disability worldwide and produces a broad spectrum of motor, sensory, cognitive, language, visual, affective, swallowing, and functional impairments [1,2]. Ranking among the leading causes of death and long-term disability worldwide, there are an estimated 12.2 million new cases and 6.55 million deaths recorded annually as of 2019 [1]. In the US alone, over 795,000 people suffer from a stroke every year, accounting for 1 in 6 deaths due to a cardiovascular disease [3]. Between 2019 and 2020, the stroke-related costs equal $56.2 billion in the US, which include health care services, treatment, and missed work [3]. A non-Hispanic black adult possesses twice the risk of having a first stroke compared to a white adult, suggesting a strong population bias in manifestation of stroke pathology [3]. Shared modifiable risk factors span both subtypes and include hypertension, the single largest contributor, accounting for over 55% of stroke-related disability-adjusted life years globally, as well as elevated body mass index, high fasting plasma glucose, ambient particulate matter exposure, and smoking [1].

This narrative review synthesizes current evidence on the relationship between stroke and olfactory function, examines the neuroanatomical substrates that may underlie post-stroke olfactory deficits, and identifies gaps in screening, rehabilitation, and post-stroke care. The available literature supports post-stroke olfactory dysfunction as an underrecognized and clinically relevant problem, but the certainty of lesion-specific associations and prevalence estimates is limited by small cohorts, heterogeneous methods, and the frequent use of case reports. Accordingly, mechanistic lesion-to-phenotype relationships are interpreted as provisional unless supported by convergent human evidence.

### Review Methodology

This article is a narrative review rather than a systematic or scoping review. For the revised review, targeted literature searches were conducted in PubMed/MEDLINE from database inception to 22 September 2026, supplemented by backward reference screening of relevant reviews and primary studies and a search of ClinicalTrials.gov for stroke-specific olfactory rehabilitation studies. Search terms were used in combinations that included stroke, cerebrovascular accident, ischemic stroke, hemorrhagic stroke, olfaction, olfactory dysfunction, smell, anosmia, hyposmia, dysosmia, odor identification, orbitofrontal cortex, insula, thalamus, piriform cortex, cerebellum, diaschisis, cognitive impairment, dementia, cerebral small vessel disease, SARS-CoV-2, COVID-19, olfactory training, oral microbiome, and periodontitis. English-language, peer-reviewed human studies were prioritized for prevalence, clinical phenotype, cognition, and rehabilitation; mechanistic, neuroimaging, animal, and non-stroke lesion studies were included when they directly informed stroke-relevant olfactory circuitry or interpretation. Case reports and small lesion series were retained for rare focal phenotypes but are identified as low-level, hypothesis-generating evidence. Articles were excluded when they were not relevant to stroke/olfaction, did not provide interpretable clinical or mechanistic information, or were non-English reports without an accessible English-language account. Because the purpose was integrative rather than exhaustive evidence grading, no PRISMA flow diagram or formal risk-of-bias meta-analysis was performed.

## 2. Stroke Pathology: Cellular and Anatomical

Broadly, stroke is classified into two pathological categories. Ischemic stroke is the most prevalent type, accounting for ~87% of all stroke cases [3]. The principal vascular etiologies include large artery atherosclerosis; cardioembolism, which is most commonly associated with atrial fibrillation; and small vessel disease, also referenced as lacunar stroke [2]. The current window for the treatment of ischemic stroke is about 4.5 h and primarily involves restoration of blood flow through the administration of tissue plasminogen activator (tPA) or use of clot retrieval devices [4]. The second category of stroke, hemorrhagic stroke, results from the rupture of a blood vessel within or around the brain parenchyma; small vessel disease is the most frequent cause of intracerebral hemorrhage, while subarachnoid hemorrhage most commonly follows rupture of an intracranial aneurysm [2].

At the cellular level, disruption of blood supply produces severe hypoxic stress in neurons which are very sensitive to this stress [5]. Within minutes of blood flow cessation, ATP depletion triggers membrane depolarization, excitotoxic glutamate release, and calcium influx. This process activates proteases and lipases that contribute to irreversible cell death through several pathways including apoptosis, necrosis, autophagy, and pyroptosis [6]. Subsequently, white matter axon fibers and astrocytes are degenerated, and the blood–brain barrier is disrupted [5]. The disruption of the blood–brain barrier leads to invasion of peripheral immune cells into the brain parenchyma, contributing to neuroinflammation and neuropathology [7]. The ischemic core contains severely injured tissue, while the surrounding tissue, known as “ischemic penumbra,” may remain functionally silent but potentially salvageable for a limited window of hours. This forms the biological rationale for time-sensitive reperfusion therapies, including intravenous thrombolysis, as well as mechanical thrombectomy [2]. Glial cells further play protective roles by limiting the expansion of the ischemic core and by active tissue remodeling through phagocytosis of the dead cells and cellular debris, angiogenesis and neurogenesis [7].

Beyond the acute lesion, stroke can also produce diaschisis, remote hypoperfusion, degeneration of connected pathways, and long-term structural remodeling. These mechanisms are especially relevant for olfaction because smell depends on distributed and recurrent networks rather than a single linear pathway. The clinical manifestations of stroke are determined largely by the location and extent of the affected vascular territory. The middle cerebral artery is the most commonly occluded vessel and may produce contralateral hemiparesis, hemisensory loss, and, with dominant hemisphere involvement, aphasia. Anterior cerebral artery infarcts affect the medial frontal and parietal lobes, whereas posterior circulation strokes, involving the vertebrobasilar system, disrupt the brainstem, cerebellum, thalamus, and occipital cortex [2]. Notably, several of these territories overlap with structures integral to olfactory processing, including the insula, orbitofrontal cortex (OFC), and thalamus, an anatomical relationship that may contribute to the vulnerability of the olfactory system following stroke.

## 3. Olfactory Dysfunction: Causes and Consequences

Despite its clinical relevance, olfactory dysfunction remains one of the most underrecognized sequelae of stroke. The sense of smell is integral to functions that directly affect patient health and quality of life, including appetite regulation and nutritional intake, detection of environmental hazards such as smoke or spoiled food, and the mediation of memory and emotional experience [8,9,10]. Anosmia and hyposmia, the complete or partial loss of smell, are estimated to affect between 3% and 20% of the general population, with neurological injury representing a significant contributing factor [11]. Olfactory dysfunction is broadly categorized as congenital and acquired; congenital dysfunction affects 0.01–0.02% of the general population [12]. Congenital anosmia can be a result of rare genetic abnormalities such as in Kallmann syndrome, characterized by deficits in embryonic migration of gonadotropin-releasing hormone (GnRH) neurons and olfactory nerves originated in the nasal placode (e.g., mutations in *KAL1*, *FGFR1,* and *PROK2* genes), or due to infection- or stress-induced events during pregnancy [13,14]. The majority of Kallmann syndrome cases are due to sporadic mutations, and familial inheritance cases are rare with 64% displaying an autosomal dominant pattern [15]. Acquired olfactory dysfunction is largely attributed to sinonasal disease, upper respiratory tract infection, traumatic brain injury or idiopathic [16]. Viral infections (e.g., COVID-19), neurodegenerative diseases and toxins are well-identified mechanisms underlying acquired olfactory dysfunction [17,18].

In the context of stroke, olfactory deficits are frequently underreported: patients are rarely screened for smell loss during acute or rehabilitation care, and many remain unaware of their own impairment [19]. However, objective assessment demonstrates that olfactory dysfunction is a measurable consequence of cerebrovascular injury, ranging from sudden anosmia following ischemic infarction of olfactory structures to subtler deficits in odor identification and hedonic appraisal [19,20]. The olfactory system’s anatomically distributed organization, spanning peripheral receptors, the olfactory bulb and tract, piriform cortex, amygdala, entorhinal cortex, insula, and OFC, renders it vulnerable to disruption across multiple stroke territories, likely contributing to the heterogeneity of deficits observed clinically.

Terminology is used consistently throughout this review: anosmia denotes complete loss of olfactory function, hyposmia denotes reduced olfactory function, and dysosmia is used as an umbrella term for qualitative distortion of odor perception (including parosmia-like or phantosmia-like phenomena when specified). Taste refers to gustatory function, whereas flavor refers to the multisensory percept arising from retronasal olfaction together with taste, oral somatosensation, and trigeminal inputs [21].

Prior SARS-CoV-2 infection is an important potential confounder in studies performed during or after the COVID-19 pandemic. COVID-19 can produce acute and persistent quantitative or qualitative olfactory dysfunction, with psychophysical testing identifying persistent deficits even when patients report subjective recovery [18,22]. Therefore, post-stroke studies should document prior COVID-19 infection, the timing of infection relative to stroke and symptom onset, the trajectory of smell recovery, and other post-viral or sinonasal causes before attributing olfactory impairment solely to the cerebrovascular event. This is especially important when a patient presents with isolated or disproportionate anosmia. Direct evidence examining the interaction among prior SARS-CoV-2 infection, stroke, and subsequent olfactory dysfunction remains limited, so this relationship should presently be treated as an unresolved confounding and effect-modification question rather than an established mechanism.

## 4. The Olfactory System and Stroke-Relevant Stages of the Olfactory Pathway

**Figure 1:**
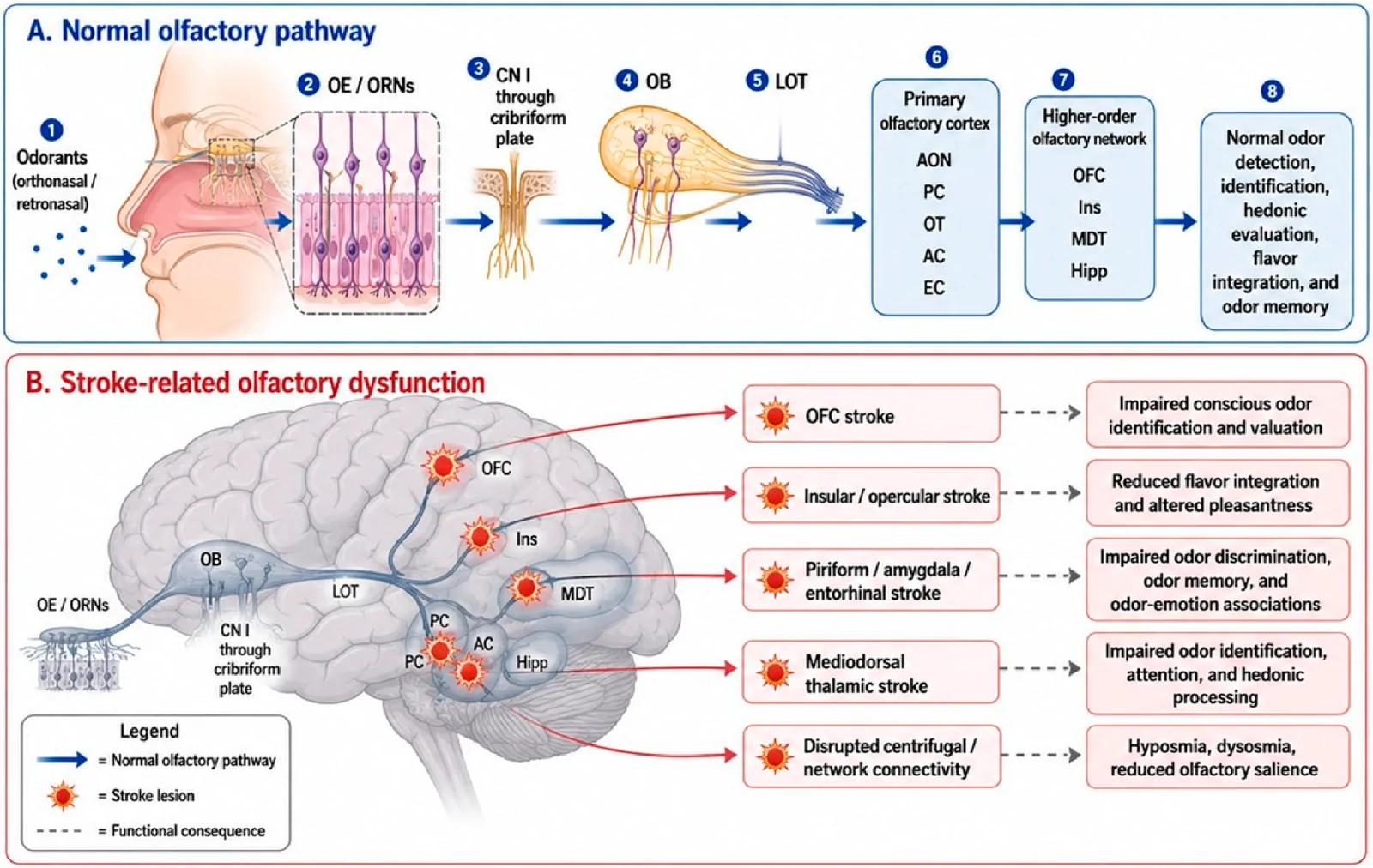
Schematic overview of the olfactory pathway and major sites where stroke can disrupt olfactory processing. In the normal pathway, odorants reach the olfactory epithelium orthonasally or retronasally, activate olfactory receptor neurons, and project via cranial nerve I to the olfactory bulb. Mitral and tufted cell output then travels through the lateral olfactory tract to primary olfactory cortical areas, including the anterior olfactory nucleus, piriform cortex, olfactory tubercle, amygdaloid complex, and entorhinal cortex, with further processing in orbitofrontal, insular, thalamic, and hippocampal networks. Stroke affecting these regions can impair odor detection, odor identification, hedonic evaluation, flavor integration, odor memory, and odor-guided behavior. Post-stroke disruption of olfactory processing may present as hyposmia, functional anosmia, impaired odor identification, altered odor pleasantness, dysosmia, reduced appetite, diminished flavor perception, impaired odor memory, or reduced odor-guided behavior. In some patients, basic odor detection may be relatively preserved while higher-order functions such as odor naming, recognition, pleasantness judgment, and flavor integration are impaired. OE—olfactory epithelium; ORNs—olfactory receptor neurons; CN I—olfactory nerve; OB—olfactory bulb; LOT—lateral olfactory tract; AON—anterior olfactory nucleus; PC—piriform cortex; OT—olfactory tubercle; AC—amygdaloid complex; EC—entorhinal cortex; OFC—orbitofrontal cortex; Ins—insula; MDT—mediodorsal thalamus; Hipp—hippocampus.

Olfaction begins when volatile odorant molecules reach the olfactory mucosa either orthonasally, during sniffing or inhalation through the nares, or retronasally, when odorants released from food and drink pass from the oral cavity through the nasopharynx during chewing, swallowing, and exhalation (Figure 1). Retronasal olfaction is especially important for flavor perception because it allows food-related volatile compounds to be integrated with taste, oral somatosensation, and trigeminal input in higher cortical regions such as the insula and OFC [21].

Both orthonasal and retronasal signals activate olfactory receptor neurons in the olfactory epithelium, a pseudostratified neuroepithelium located in the superior nasal cavity. This epithelium contains bipolar olfactory receptor neurons, sustentacular supporting cells, and basal stem cells. Odorant receptors are G protein-coupled receptors expressed on the cilia of olfactory receptor neurons, where odorant binding initiates transduction and generates activity in cranial nerve I [23,24,25]. The olfactory pathway begins in the olfactory epithelium, with olfactory receptor neuron axons crossing the cribriform plate and projecting ipsilaterally to the olfactory bulb, where they synapse in glomeruli [24,25].

The olfactory bulb is the first central relay of the main olfactory system (Figure 1). Axons from olfactory receptor neurons converge onto olfactory bulb glomeruli, where they contact mitral and tufted projection neurons as well as juxtaglomerular interneurons, including periglomerular cells, short-axon cells, and external tufted cells [26,27]. This glomerular organization allows odor information to be transformed from peripheral receptor activation into distributed spatial and temporal patterns of bulbar activity. Periglomerular and short-axon cells provide local and interglomerular inhibition, often using GABA and dopamine, while external tufted cells are glutamatergic and can exhibit rhythmic bursting that helps coordinate glomerular output [27,28]. Olfactory sensory neuron terminals contact mitral cells, external tufted cells, periglomerular cells, and short-axon cells, with dopaminergic/GABAergic short-axon cells forming oligoglomerular or polyglomerular networks that support interglomerular modulation [29].

These inhibitory and excitatory microcircuits are relevant to stroke because the olfactory bulb does not function only as a passive relay. It receives extensive centrifugal input from olfactory cortex, frontal cortex, hippocampal structures, cholinergic basal forebrain, locus coeruleus, raphe nuclei, and GABAergic systems, which shape odor responses, attention, salience, and experience-dependent modulation [25,30]. Therefore, vascular injury to frontal, limbic, or neuromodulatory systems may alter bulbar gain, odor sensitivity, attention to odors, or odor learning even when the olfactory epithelium and bulb are structurally intact.

Mitral and tufted cell axons leave the olfactory bulb through the lateral olfactory tract and project directly to primary olfactory cortical and limbic structures. Primary olfactory cortex includes the anterior olfactory nucleus, olfactory tubercle, piriform cortex, cortical and periamygdaloid amygdala-related regions, nucleus of the lateral olfactory tract, and parts of the entorhinal cortex [25,31]. The piriform cortex is the largest primary olfactory cortical area and plays a central role in odor identity, odor object formation, olfactory learning, and associative memory [25,32].

A distinctive feature of olfaction is that early cortical processing does not require a mandatory thalamic relay before reaching primary cortex, unlike vision, audition, and somatosensation [33]. This direct bulb-to-cortex organization may help explain why focal cortical and limbic lesions can produce selective deficits in odor identification, discrimination, memory, or hedonic appraisal rather than a simple loss of detection threshold. Human connectivity work also shows that primary olfactory subregions form distinct large-scale functional networks, with the anterior olfactory nucleus, olfactory tubercle, and frontal and temporal piriform cortices showing separable whole-brain connectivity patterns [34].

After primary olfactory cortex, odor information is distributed to secondary and multimodal regions, especially the OFC, insula, amygdala, hippocampal formation, hypothalamus, cingulate cortex, and thalamus [35,36]. The OFC is a major higher-order target for conscious odor perception, odor identification, reward value, pleasantness, and integration of smell with taste and oral texture [35,37]. The insula is also strongly implicated in olfaction and flavor because it participates in multisensory, affective, interoceptive, and gustatory processing. Tractography, primate tracing, functional imaging, and lesion evidence support connections between primary olfactory cortex and the insula, and insular lesions can produce olfactory disturbances, particularly altered pleasantness, subjective smell changes, or difficulty distinguishing odors [38]. This is important for stroke because the insula is commonly involved in middle cerebral artery territory infarcts. Thus, insular stroke may contribute to altered flavor, odor unpleasantness, distorted odor quality, reduced food enjoyment, or reduced appetite, even when basic odor detection is preserved [38,39,40].

Although olfaction bypasses the thalamus on its way to primary olfactory cortex, the mediodorsal thalamus remains important for higher-order olfactory processing. The mediodorsal thalamic nucleus receives input from primary olfactory regions, including piriform cortex, olfactory tubercle, amygdala, lateral entorhinal cortex, and anterior olfactory nucleus, and has reciprocal connections with OFC and agranular insular regions [33]. Thalamic involvement is particularly relevant to stroke phenotypes. Patients with focal thalamic lesions may show relatively spared odor detection but impaired odor identification and altered hedonic ratings, especially reduced pleasantness for pleasant odors [41]. Case reports of mediodorsal or bilateral thalamic infarction similarly describe disturbances of smell, taste, food intake, and odor pleasantness [42,43]. Okamoto et al. also reported that acute ischemic stroke patients without MRI-visible thalamic lesions but with thalamic hypoperfusion showed reduced odor identification and recognition, suggesting that diaschisis or remote perfusion changes may affect olfactory behavior even when the visible infarct is outside the classic olfactory pathway [44]. These observations support a model in which thalamic injury preferentially affects attention-dependent, semantic, affective, and evaluative dimensions of olfaction rather than peripheral odor detection.

## 5. Direct and Indirect Effects of Stroke on Olfactory Processing

Stroke may affect the olfactory system directly, indirectly, or through a combination of mechanisms. Direct effects occur when the lesion involves olfactory pathway structures themselves, such as the anterior olfactory region, olfactory tract, piriform cortex, amygdala, entorhinal cortex, OFC, insula, or thalamus [38,41,45]. Indirect effects occur when stroke affects regions that modulate olfactory processing, disrupts distributed networks, alters perfusion in connected areas, impairs cognition or language required for odor identification, or changes respiratory and sniffing behavior [33,34,44,46].

This distinction is clinically important because a patient does not need to have an infarct in the olfactory bulb or primary olfactory cortex to develop olfactory symptoms. A frontal stroke may impair conscious odor recognition and valuation. A thalamic stroke may impair odor identification or pleasantness. An insular stroke may alter flavor integration or odor affect (hedonic value) as the insular cortex (insula) acts as a critical multisensory hub where taste, smell, and internal bodily sensations converge to create our perception of flavor. A cerebellar stroke may change sniff dynamics and olfactomotor control. A large hemispheric stroke may disrupt attention, naming, memory, or awareness, thereby affecting performance on odor identification tasks [37,38,41,46]. In addition, diaschisis or remote hypoperfusion may impair olfactory behavior even when the visible infarct is outside classical olfactory structures [44].

From a stroke perspective, olfactory dysfunction can therefore arise at several levels. Damage to inferior frontal or orbitofrontal regions may impair conscious odor recognition, valuation, and flavor-related reward [35,37]. Medial temporal or limbic infarcts involving piriform cortex, amygdala, hippocampus, or entorhinal cortex may affect odor identity, odor memory, odor–emotion associations, and learned odor meaning [9,25,32]. Insular and opercular strokes may disturb multisensory flavor integration and the affective or interoceptive experience of odors [38,39,40]. Thalamic strokes, especially involving mediodorsal regions, may impair odor identification and pleasantness while leaving basic detection relatively intact [41,42,43]. Finally, disruption of fronto-limbic, thalamo-cortical, or centrifugal feedback circuits may alter olfactory attention, salience, and odor-guided behavior [30,33,44]. Clinically, this means that post-stroke olfactory dysfunction may present not only as anosmia or hyposmia, but also as impaired odor naming, reduced food enjoyment, altered pleasantness, dysosmia, impaired flavor perception, impaired odor memory, or reduced awareness of the deficit [19,43,47].

## 6. How Common Is Post-Stroke Olfactory Dysfunction?

The true prevalence of post-stroke olfactory dysfunction remains uncertain, and the available studies should not be treated as yielding a single pooled estimate. Published estimates differ because investigators have used self-report questionnaires, bedside interviews, psychophysical smell tests, neurophysiological methods, acute and chronic cohorts, rehabilitation samples, and case–control designs [19,47,48,49]. Differences in age, stroke severity, lesion distribution, time since stroke, cognitive-language status, and ascertainment of non-stroke causes of smell loss further limit direct comparison. The principal human studies are summarized in Table 1.

Objective testing appears to detect more dysfunction than self-report alone. In a prospective cohort of 78 chronic stroke patients tested one year after stroke admission, Wehling and colleagues found 28.2% hyposmia and 15.4% olfactory loss, including functional and complete anosmia [19]. Age and the National Institutes of Health Stroke Scale score were predictors of olfactory dysfunction, and patients with olfactory dysfunction rated odor items as less pleasant [19]. Importantly, self-reported olfactory function did not reliably distinguish patients with normal olfaction from those with reduced olfactory performance, suggesting that routine clinical questioning may miss many patients with meaningful olfactory impairment [19].

Bedside questionnaire studies similarly suggest that smell and taste dysfunction occur in both acute and chronic stroke populations. Schön and colleagues reported subjective smell dysfunction in approximately 12% of stroke patients and taste dysfunction in approximately 38%, emphasizing that routine bedside assessment is feasible but should be validated against objective testing [48]. Case–control psychophysical work has reported worse smell identification in patients with stroke than in healthy controls, along with concurrent taste impairment and reduced food liking [47]. Such findings are valuable because they connect olfactory dysfunction to patient-centered outcomes such as appetite and food enjoyment; however, they should be interpreted in light of sampling, since rehabilitation or clinic populations may overrepresent more symptomatic patients.

Taken together, these heterogeneous human studies show that measurable olfactory abnormalities can occur after stroke and may persist into the chronic phase, but they do not justify a single prevalence estimate. Objective testing can identify abnormalities not captured by self-report, whereas questionnaire-based studies may better capture patient-perceived taste, flavor, appetite, and quality-of-life consequences [19,47,48,49]. Larger prospective cohorts using common case definitions and standardized testing at comparable post-stroke intervals are needed before population-level prevalence can be estimated with confidence.

## 7. What Does Olfactory Dysfunction Look Like After Stroke?

Post-stroke olfactory deficits are heterogeneous and may involve sensitivity, identification, discrimination, hedonic evaluation, flavor integration, memory, or qualitative odor perception. For this reason, “smell loss” is an imprecise umbrella term.

One common pattern is reduced sensitivity, or threshold elevation, which may manifest as hyposmia or anosmia. In some patients, this may be lateralized, particularly if the lesion affects unilateral olfactory structures or olfactomotor pathways [19,46]. A second pattern is impaired odor identification or discrimination. These patients may detect that an odor is present but cannot name it, recognize it, or distinguish it from similar odors. This phenotype may reflect central olfactory network injury, but it may also be influenced by aphasia, semantic impairment, memory dysfunction, attention deficits, or executive dysfunction [19,44,49].

A third pattern is hedonic or affective distortion. Patients may report that odors have lost their pleasant character, smell unpleasant, or no longer contribute normally to food enjoyment. This phenotype is particularly important because it may drive appetite changes, reduced oral intake, weight loss, or maladaptive dietary compensation. Classic thalamic lesion reports show that odor identification may be preserved while pleasantness is selectively impaired, demonstrating that hedonic olfaction can dissociate from basic detection [41,43]. Wehling and colleagues similarly found altered pleasantness ratings in chronic stroke patients with olfactory dysfunction [19].

A fourth pattern includes qualitative phenomena such as dysosmia, parosmia-like distortions, or phantosmia-like complaints. These symptoms are less commonly discussed in stroke than in post-viral or traumatic etiologies, but insular and temporal network involvement may plausibly contribute to altered odor quality, unpleasant odor bias, or olfactory hallucinations. Beume and colleagues described olfactory hallucinations as a primary symptom of ischemia in the right posterior insula, supporting the possibility that focal ischemia can produce qualitative olfactory phenomena [38,51].

Finally, patients may experience reduced flavor perception even when they describe the issue as a “taste” problem rather than a smell problem. Because flavor depends heavily on retronasal olfaction, stroke-related disruption of olfactory, gustatory, insular, orbitofrontal, and somatosensory integration may cause food to seem bland, unpleasant, or less rewarding [21,39,47].

## 8. Lesion Locations That Matter: What Do We Learn from Animal and Case Studies?

Because olfaction depends on distributed networks, post-stroke olfactory dysfunction cannot be assigned reliably to a single lesion site. Several stroke-relevant nodes have been associated with particular olfactory phenotypes, but the strength of evidence varies considerably. Some associations derive from small lesion cohorts, isolated case reports, non-stroke focal lesions, or mechanistic animal studies. Accordingly, the lesion-to-phenotype relationships below should be interpreted as biologically plausible and hypothesis-generating rather than deterministic clinical rules. Table 2 summarizes both the reported phenotype and the level/limitations of supporting evidence.

### 8.1. Anterior Olfactory Skull-Base Region and Olfactory Tract

Sudden isolated anosmia can, in rare cases, be a cerebrovascular presentation. Ischemic infarcts involving the anterior olfactory skull-base region adjacent to the olfactory sulci and straight gyri, including the olfactory tract distribution, may produce acute smell loss and mimic more common causes such as viral anosmia. Theodorou and colleagues described CT- and MRI-visible bilateral ischemic lesions adjacent to the olfactory sulci in a patient with anosmia, supporting ischemic olfactory infarction as a vascular cause of sudden smell loss [45]. This is clinically relevant since the patient had a history of type-2 diabetes and gastritis and presented with symptoms mimicking potential coronavirus infection. Thus, sudden anosmia may be attributed to infection, sinonasal disease, or post-viral dysfunction, potentially delaying consideration of vascular causes in appropriate contexts.

### 8.2. Insula and Opercular/Frontoparietal Cortex

The insula is a multisensory integration hub tightly tied to flavor, taste, oral somatosensation, interoception, and affective response. Although the OFC can be the primary center for integrating and processing of taste and smell inputs, the insula and opercular or frontoparietal cortex receive taste afferents from the nucleus solitarius and thalamus. Thus, lesions involving insula and opercular/frontoparietal cortex can disrupt integration of taste and smell signals in the brain [38,39,40]. In addition, left angular gyrus and inferior parietal gyrus in the parietal lobe are involved in olfactory working memory [52]. Functional MRI studies reveal that these areas are highly activated during processing of olfactory working memory [52]. Therefore, these areas are critical for olfactory perception, integration and memory.

Moo and Wityk described olfactory and taste dysfunction after bilateral middle cerebral artery strokes involving posterior insula and opercular/frontoparietal cortex relevant to multisensory integration [40]. Because symptoms became apparent after a second contralateral stroke in a single patient, the report raises the possibility that bilateral network injury can unmask or amplify chemosensory dysfunction, but it does not establish that bilateral lesions are required. The insula is frequently affected in middle cerebral artery stroke and may therefore contribute to changes in food enjoyment, appetite, flavor perception, or odor affect. Standard threshold or identification tests may miss such higher-order complaints: patients can detect odors but experience them as distorted, unpleasant, weak, or poorly integrated with taste [38]. A reported left insular cavernoma similarly altered odor intensity and valence, but this non-stroke case should be viewed as mechanistic support rather than direct evidence of a stroke-specific lateralization rule [38,53].

Heightened sensitivity to unpleasant odors and olfactory hallucinations have also been described after insular lesions [39,51]. Mak and colleagues reported altered suprathreshold odor and taste intensity after a left posterior insular stroke, with prominent contralateral intensity changes [39]. Some small studies have proposed hemispheric asymmetries in affective processing, including relatively greater right-hemisphere contributions to pleasant stimuli and left-hemisphere contributions to unpleasant stimuli [41]. However, the published insular literature is too small and heterogeneous to conclude that left- and right-sided lesions produce reliably distinct olfactory syndromes. Laterality should therefore be recorded and tested prospectively rather than inferred from individual cases.

### 8.3. Thalamus

Although olfaction is often construed as bypassing the thalamus, multiple lines of evidence in rodent models using electrophysiology, behavior, and immunohistochemistry approaches show that thalamic nucleus, especially mediodorsal thalamus, known for its role in higher order cognitive function, contributes to experience-dependent, modality-specific olfactory functions including olfactory attention, discrimination, and hedonic processing, as well as odor-guided decision making [33,54]. As such, mediodorsal thalamus receives projection from piriform cortex and gustatory cortex and is connected reciprocally with OFC. A novel odor, compared with a familiar odor, leads to greater neuronal activation determined by c-Fos reactivity, whereas a familiar taste or odor–taste mixture results in greater neuronal activation in the mediodorsal thalamus [54]. In addition, the preference for a familiar odor over a novel odor (such as preference for water over isoamyl acetate-odorized water) is eliminated when the novel odor is coupled with a novel taste (such as isoamyl acetate-odorized water with sucrose) over several days, suggesting an experience-driven integration of behaviorally relevant chemosensory stimuli including odor neophobia, potentially involving mediodorsal thalamus and the higher-order structures in food-related behavior [54]. The mediodorsal thalamus enhances neural coupling with piriform cortex during odor sampling and with OFC during decision making during two-alternative odor discrimination tasks [33]. Specifically, the mediodorsal thalamus is likely to be spared for sensory thalamic relay relevant to odor encoding, and state-dependent modulation, but remains an important node for sensorimotor representation of odor-guided, goal-directed behavior [33,55].

Human thalamic lesion studies further support a role for the mediodorsal thalamus in higher-order olfaction, although the available samples are small. Case reports describe persistent changes in odor/taste pleasantness, appetite, and weight after dorsomedial or bilateral thalamic infarction [42,43]. In a lesion study of 17 patients and 18 age-matched controls, thalamic lesions impaired odor identification while largely sparing basic detection; right-sided lesions were associated with reduced pleasantness ratings for pleasant odors, and both left- and right-sided groups showed reduced sniff vigor [41]. These findings suggest, but do not prove, lateralized contributions to hedonic processing. In addition, 19 acute ischemic stroke patients without MRI-visible thalamic infarction but with thalamic hypoperfusion on SPECT had lower odor identification and recognition performance than 9 stroke controls without thalamic hypoperfusion, despite similar absolute detection thresholds [44]. This latter finding is consistent with diaschisis or remote network dysfunction and argues against assuming that only the visible infarct determines the olfactory phenotype.

### 8.4. OFC and Frontal Regions

The OFC is a major secondary olfactory region that receives input from primary olfactory cortex, including piriform cortex [56,57]. Human functional neuroimaging implicates rostral OFC in higher-order olfactory processing [35]. A single traumatic brain injury case combining fMRI and autonomic recording found absent conscious olfaction despite preserved odor-evoked activity in the contralateral OFC, supporting a role for right OFC in conscious olfactory perception [37]. Because this evidence comes from a non-stroke single-lesion case, it should not be generalized as a deterministic right-hemisphere rule for stroke. More broadly, OFC injury may plausibly affect odor identification, valuation, flavor reward, and conscious awareness, while superior, middle, and inferior frontal regions and motor areas are also recruited during olfactory working-memory tasks [36,52].

### 8.5. Medial Temporal and Limbic Structures

The piriform cortex, amygdala, entorhinal cortex, and hippocampus support odor identity, associative learning, odor memory, and odor–emotion links [9,25,32]. Because odors are strongly tied to autobiographical memory and affect, which involve the amygdala-hippocampus as well as medial OFC and posterior cingulate cortex, damage to limbic olfactory structures may have psychological and quality-of-life consequences that are not captured by threshold testing alone [9,58]. Matsunaga and colleagues report that odor-evoked specific memories generate 6.5 times more nostalgia than odors that do not elicit a specific memory or an event [59]. Reid and colleagues further observe that nostalgic odors bring three times more positive than negative emotion [60]. In fact, odor-evoked nostalgia is more powerful than music-evoked nostalgia [61]. Matsunaga and colleagues further note that plasma levels of proinflammatory cytokines such as tumor necrosis factor α (TNF-α) and interferon-γ (IFN-γ) are significantly reduced following odor experience evoking positive emotions and autobiographical memory, implicating odor-evoked emotions in modulating neuroimmune responses [59]. Both medial OFC and posterior cingulate cortex are activated by odor-evoked autobiographical memory, as observed by PET imaging—further emphasizing the role for limbic system in olfaction. Thus, stroke involving the limbic system may impair odor recognition, odor memory, emotional associations with odors, and learned odor meaning, which in turn may critically impact both psychological and physiological well-being.

### 8.6. Cerebellum and Olfactomotor Control

Although the cerebellum is not traditionally considered part of the core olfactory pathway, fMRI studies show cerebellar activation during odor sampling and sniffing [62]. Sniff dynamics influence odorant delivery to the olfactory mucosa and therefore shape sensory input. Small lesion studies, largely outside a stroke-specific cohort, have reported nostril-specific olfactory impairments after unilateral cerebellar injury and impaired identification in cerebellar degeneration [46,63]. These observations support an olfactomotor contribution but remain indirect evidence for stroke. In a patient with cerebellar stroke, altered breathing, reduced sniff vigor, facial weakness, or impaired sensorimotor coordination could plausibly degrade odor sampling even when peripheral receptor neurons are intact; direct stroke-specific testing is needed to establish how often this mechanism is clinically important.

### 8.7. Laterality, Diaschisis, and Network-Level Interpretation

Across the available literature, laterality findings are suggestive but not yet sufficiently consistent for bedside prediction. Right orbitofrontal and right thalamic involvement has been linked to aspects of conscious or hedonic odor processing [37,41], whereas several insular reports involve left-sided lesions [38,39]; bilateral middle cerebral artery injury has also been associated with combined smell and taste dysfunction [40]. These observations come from small cohorts or individual cases and may be influenced by task demands, lesion extent, and publication bias. Moreover, stroke can disrupt olfaction without directly injuring a canonical olfactory node. Thalamic hypoperfusion remote from the visible infarct [44], interruption of fronto-limbic or thalamo-cortical connections, and altered centrifugal feedback provide mechanisms by which diaschisis and network disconnection may generate similar phenotypes from different lesion locations. A network model therefore predicts probabilistic associations, not one-to-one mappings: lesion side and location should be integrated with perfusion/connectivity, cognitive-language status, and multidimensional olfactory testing.

**Table 2:** Lesion- and network-level associations with olfactory phenotypes after stroke. The table summarizes reported associations and the limitations of the supporting evidence; it should not be read as a deterministic lesion-localization map.

Lesion/Network	Reported Phenotype	Evidence Base	Interpretation/Limitation
Anterior olfactory region/olfactory tract	Acute anosmia or marked sensitivity loss	Rare ischemic case reports [45]	Direct focal vascular injury is plausible, but incidence and generalizability are unknown.
Insula/operculum	Altered odor/taste intensity, flavor integration, pleasantness; qualitative symptoms	Small stroke reports and non-stroke lesion cases [38,39,40,51]	Multisensory/affective role is plausible; laterality claims remain uncertain.
Mediodorsal thalamus/thalamic network	Identification and hedonic deficits with relatively preserved detection; altered sniff vigor	Small lesion cohort, case reports, and perfusion study [41,42,43,44]	Convergent evidence supports higher-order involvement; diaschisis may mimic direct thalamic injury.
Orbitofrontal/frontal regions	Conscious odor identification, valuation, reward, working memory	Functional imaging plus non-stroke focal lesion evidence [35,36,37,52]	Stroke-specific lesion-symptom evidence is limited; right-sided specialization is not established as a clinical rule.
Piriform/amygdala/entorhinal/hippocampal network	Odor identity, discrimination, memory, odor–emotion associations	Mechanistic, imaging, and broader lesion literature [9,25,32,58]	Strong network plausibility but limited stroke-specific focal-lesion cohorts.
Cerebellar/olfactomotor circuits	Sniff-related changes, nostril-specific detection/identification deficits	Small lesion studies and cerebellar degeneration data [46,62,63]	Supports olfactomotor mechanism; direct stroke-specific evidence remains sparse.
Diaschisis/distributed disconnection	Variable hyposmia, impaired identification, hedonic or attentional changes	Perfusion and network evidence [30,33,44,64,65,66]	Different lesions may converge on a shared network phenotype; requires prospective connectivity/lesion mapping.

## 9. Secondary Degeneration and Structural Remodeling After Stroke

Beyond the location of the infarct, stroke can drive longer-term remodeling in olfactory structures. In an MRI case–control study of 41 patients with stroke and 41 controls, Kültür and colleagues measured peripheral olfactory structures, including olfactory bulb volume and olfactory sulcus depth, as well as central regions such as the insula and amygdala [50]. They reported smaller peripheral and central smell-related regions in patients with stroke, and left olfactory bulb volume decreased with longer stroke duration [50]. These findings suggest that post-stroke olfactory dysfunction may involve ongoing degeneration or inflammation-associated change rather than simple acute injury followed by recovery.

This aligns with a broader model in which post-stroke olfactory dysfunction reflects a mixture of direct circuit injury, remote network dysfunction, diaschisis, hypoperfusion, and chronic structural change. Such mechanisms may explain why some patients continue to experience olfactory impairment long after the acute lesion and why olfactory deficits may not map precisely onto the visible infarct alone [44,50].

Although long-term recovery of olfactory function remains considerably unknown, available documentation indicates that olfactory dysfunction may extend well beyond the acute phase, with impairment persisting and remaining measurable within a significant number of patients following up to one year after stroke [19]. Restoration may differ due to the underlying mechanics of impairment; dysfunction sourced from transient network-level disturbances likely alter from those triggered by direct trauma to olfactory structures or structural modification [44,45,50]. Regardless, the determinants that anticipate recovery continue to be unexplored, and longitudinal studies monitoring olfactory effects after stroke are understudied. Additional research is essential for understanding whether post-stroke olfactory dysfunction adheres to predictable recovery patterns.

## 10. Why Olfaction Matters Clinically After Stroke

Smell loss is not merely a sensory curiosity. It can affect patient outcomes. First, olfactory dysfunction can affect nutrition and appetite. Loss or distortion of smell reduces food enjoyment and may contribute to reduced oral intake, weight loss, or poor nutritional status [8,20,43,47]. Some patients may compensate by adding excessive salt or sugar to food, which may complicate management of hypertension, diabetes, or vascular risk factors. Others may lose interest in eating altogether, reducing energy intake during a period when nutrition is important for rehabilitation and recovery.

Second, olfactory dysfunction creates safety risks. Patients with impaired smell may fail to detect smoke, gas leaks, spoiled food, chemicals, or other environmental hazards [10,11]. Because self-report is unreliable, patients may not spontaneously disclose smell loss and may not receive appropriate counseling unless clinicians ask directly and test objectively [19].

Third, olfactory dysfunction may affect mood, quality of life, memory, and social behavior. Odor hedonics are tightly linked to emotion and memory circuits [9,36]. Reduced pleasantness, distorted odor perception, or loss of odor-evoked memory may contribute to diminished pleasure, social withdrawal, anxiety, or depressive symptoms. Wehling and colleagues found altered pleasantness ratings in chronic stroke patients with olfactory impairment, supporting the relevance of affective olfactory changes after stroke [19].

Fourth, olfactory dysfunction may exacerbate an inflammatory response. Inflammation is often identified as an underlying condition in several systemic, peripheral as well as neurological, neuropsychiatric and neurodegenerative diseases. Odor-evoked memories may mitigate inflammation through psychoneuroimmune interactions [59]. Plasma level of IFN-γ is negatively correlated with the activation of medial OFC and posterior cingulate cortex, areas associated with odor-evoked autobiographical memory.

**Figure 2:**
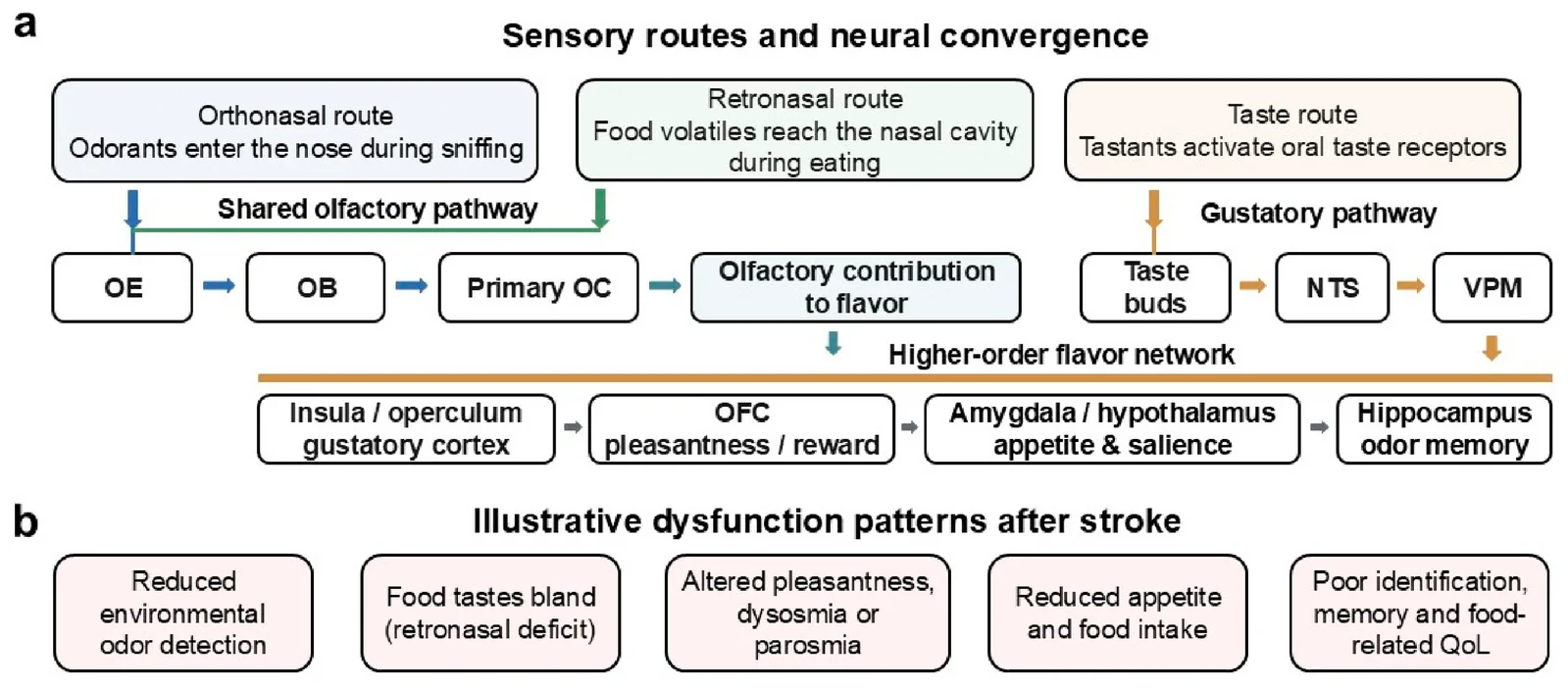
Orthonasal and retronasal olfaction, taste pathways and flavor dysfunction after stroke. (**a**) Odorants reach the olfactory system through two principal routes. In orthonasal olfaction, odorants enter the nasal cavity during sniffing. In retronasal olfaction, food volatiles released during eating pass from the oral cavity to the nasal cavity and stimulate the same olfactory apparatus. Thus, both orthonasal and retronasal odorants reach the olfactory epithelium (OE) and then follow the shared olfactory pathway through the olfactory bulb (OB) and primary olfactory cortex. In parallel, taste information travels from taste buds to the nucleus of the solitary tract (NTS), then to the ventral posteromedial thalamic nucleus (VPM), and onward to the insula/operculum, where gustatory processing contributes to flavor perception. Olfactory and gustatory information converge in higher-order flavor networks involving the insula/operculum, orbitofrontal cortex (OFC), amygdala, hypothalamus, and hippocampus. Green and blue arrows indicate olfactory pathways, brown arrows indicate gustatory pathways. (**b**) Stroke affecting these pathways may cause reduced environmental odor detection, impaired retronasal flavor perception such that food “tastes bland,” altered pleasantness, dysosmia or parosmia, reduced appetite, and impaired food-related quality of life. NTS—nucleus of the solitary tract; OB—olfactory bulb; OE—olfactory epithelium; OFC—orbitofrontal cortex; Primary OC—primary olfactory cortex; QoL—quality of life; VPM—ventral posteromedial thalamic nucleus.

Fifth, olfactory dysfunction may indirectly affect rehabilitation engagement. Poor appetite, reduced enjoyment, fatigue related to inadequate nutrition, and mood changes may reduce motivation and participation in therapy. Therefore, olfactory dysfunction should be considered part of the broader post-stroke recovery environment rather than an isolated sensory deficit (Figure 2).

While current findings are inadequate to determine olfactory dysfunction as a prognostic biomarker, these findings suggest that olfactory assessment may provide insight outside of the presence or absence of sensory impairment alone. Considering the widespread nature of olfactory processing and its perceived relationship with stroke severity and network dysfunction, olfactory deficits may provide information into broader neurological consequences of cerebrovascular injury. Future studies should consider investigating whether post-stroke olfactory dysfunction is aligned with cognitive outcomes, rehabilitation engagement, nutritional status, or long-term functional recovery. If such relationships exist, olfactory assessment could present as an underutilized tool for identifying patients who may benefit beyond conventional measures of stroke severity.

## 11. Measurement: Why the Problem Is Underestimated

One major reason post-stroke olfactory dysfunction is underestimated is measurement difficulty. Self-report is often unreliable: patients may not notice the deficit, may attribute it to aging or congestion, or may describe the problem as reduced taste, appetite, or flavor. Aphasia, memory impairment, inattention, neglect, executive dysfunction, reduced awareness, visual deficits, or competing neurological symptoms can also affect both symptom reporting and performance on odor-identification tasks. Consequently, post-stroke assessment should distinguish a primary olfactory deficit from limitations imposed by language, cognition, attention, or response format.

In Wehling’s chronic stroke cohort, self-reported olfactory function did not separate patients with normal olfaction from those with objective impairment [19]. Objective psychophysical tests are therefore essential. Practical tools include the University of Pennsylvania Smell Identification Test and Sniffin’ Sticks [67,68,69]. Sniffin’ Sticks is especially useful because it can separately assess threshold, discrimination, and identification, often summarized as a TDI score [68,69]. This separation matters because stroke may impair one domain while sparing another. For example, thalamic or frontal lesions may affect identification or pleasantness more than detection threshold [41].

Testing should be adapted to the patient’s neurological profile. When aphasia or semantic retrieval is prominent, threshold and discrimination tasks can reduce naming demands, and identification can use forced-choice pointing or picture matching rather than free naming. Instructions should be brief, demonstrated with practice items, and comprehension should be confirmed before scoring. For patients with inattention or fatigue, shorter blocks with rest periods may improve reliability. In neglect or visual-field impairment, response alternatives should be placed centrally or delivered in an auditory/nonvisual format when feasible. Memory-dependent tasks should be interpreted alongside formal memory testing, and cognitive, language, attentional, and perceptual status, as well as any accommodations, should be documented so that an apparent odor-identification deficit is not automatically attributed to olfactory network injury.

Hedonic measures should be added, when possible, particularly if patients complain that food is unpleasant, smells are distorted, or “everything smells wrong” while standard smell scores appear normal. This is particularly relevant for insular and thalamic lesions, where hedonic or affective olfactory processing may be disrupted despite preserved basic detection [38,41,43]. Retronasal olfaction and flavor-related testing may also be important because many patients experience the functional problem during eating rather than during isolated odor sniffing [21,47].

Lateralized testing may be helpful in selected patients because stroke effects may be asymmetric. Nostril-specific testing can reveal deficits that bilateral testing may obscure, especially when olfactomotor or unilateral pathway involvement is suspected [46]. Research settings may also use olfactory event-related potentials or neuroimaging-based approaches to detect objective abnormalities even when subjective perception appears preserved. Cecchini and colleagues highlighted the potential value of olfactory testing and neurophysiological approaches in patients with ischemic stroke [49].

**Figure 3:**
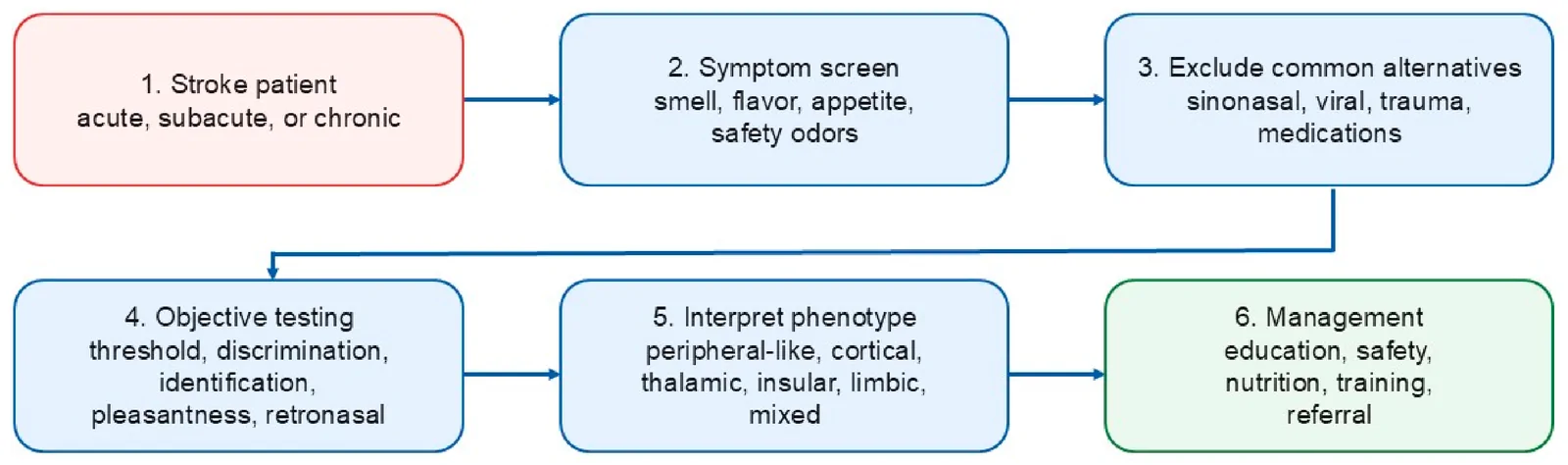
Proposed clinical screening and testing algorithm for olfactory dysfunction after stroke. Because olfactory dysfunction after stroke may be underrecognized, clinical evaluation should include both symptom screening and objective testing. Patients should be asked about smell loss, distorted odors, reduced food enjoyment, reduced appetite, impaired detection of smoke or spoiled food, and changes in flavor perception. Non-stroke causes of olfactory dysfunction, including sinonasal disease, viral illness, head trauma, medication effects, and neurodegenerative disease, should be considered. Objective testing can then separate odor threshold (odor sensitivity), discrimination (same vs. different odor judgement), identification (odor naming or recognition), pleasantness, retronasal olfaction (odor perception through the mouth/nasopharynx), and odor memory. The resulting profile may help distinguish peripheral-like smell loss from central deficits involving orbitofrontal, insular, limbic, thalamic, or network-level dysfunction. Management may include patient education, safety counseling, nutrition support, olfactory training, follow-up testing, and referral when appropriate.

A multidimensional post-stroke olfactory assessment framework should therefore include odor threshold, discrimination, identification, pleasantness, retronasal olfaction, taste, flavor perception, appetite, food liking, nutrition, mood, cognition, language, and quality of life (Figure 3). The steps include screening: initial symptom questions used to identify possible olfactory dysfunction; psychophysical testing: behavioral smell testing using standardized odor stimuli; threshold: lowest concentration at which an odor can be detected; discrimination: ability to tell whether odors are the same or different; identification: ability to name or select the correct label for an odor; pleasantness rating: judgment of how pleasant or unpleasant an odor is; retronasal testing: testing odor perception through the mouth/nasopharynx, relevant to flavor; peripheral-like pattern: pattern dominated by reduced odor sensitivity; and central pattern: pattern dominated by impaired identification, memory, pleasantness, attention, or flavor integration.

## 12. Management and Rehabilitation

There is currently no stroke-specific, universally accepted treatment pathway for olfactory loss comparable to dysphagia, aphasia, or motor rehabilitation pathways. However, several management steps are clinically defensible.

First, clinicians should evaluate non-stroke contributors that are common in stroke survivors, including sinonasal disease, allergies, smoking, xerostomia, medication effects, neurodegenerative disease, and recent viral infections [11,70]. For patients evaluated during or after the COVID-19 era, prior SARS-CoV-2 infection and the timing of smell symptoms relative to infection and stroke should be documented because persistent post-viral dysfunction can confound causal attribution [18,22]. Identifying these factors is important because some are treatable and others materially change interpretation of olfactory testing.

Second, safety counseling should be provided when dysfunction is present. Patients should be advised about smoke and gas detectors, food spoilage strategies, labeling food dates, using visual or caregiver checks, and avoiding reliance on smell alone for safety decisions. This recommendation is supported by the broader olfactory-disorder literature showing that smell loss affects personal safety and well-being [10].

Third, nutrition and appetite should be assessed. Patients with reduced food enjoyment, weight loss, or poor intake may benefit from dietary counseling, flavor enhancement strategies, texture modification, or involvement of nutrition specialists. Because some patients compensate with excess salt or sugar, counseling should also consider vascular risk management [8,47].

Fourth, olfactory training may be considered, but the evidentiary basis should be separated by etiology. Meta-analytic evidence indicates that repeated structured odor exposure can improve several olfactory outcomes across mixed, predominantly non-stroke causes of olfactory dysfunction [71]. Those findings support biological plausibility and clinical feasibility, but they do not establish efficacy after stroke because central network injury, aphasia, cognitive impairment, and motor/sensory comorbidities may alter both treatment delivery and outcome measurement. Stroke-specific randomized evidence is therefore still insufficient to recommend a standardized protocol.

Stroke-specific interventional evidence remains limited but is emerging. RE-OLF (NCT04703218), a registered study of olfactory re-education after cerebrovascular accident, is an important step toward stroke-specific evidence and is cited here from the primary ClinicalTrials.gov registry [72]. The broader PIONEER review also underscores the limited evidence base for interventions targeting perceptual disorders after stroke [73]. Until stroke-specific results are available, olfactory training should be presented as a reasonable individualized option extrapolated from other etiologies rather than as an established post-stroke treatment. Current care should continue to emphasize identification of reversible contributors, safety counseling, nutrition support, follow-up testing, and referral to neurology or otolaryngology when appropriate.

## 13. Olfaction as a Marker of Broader Cerebrovascular, Cognitive, and Aging Risk

Emerging evidence also suggests that olfactory dysfunction may not only follow stroke but may also relate to broader cerebrovascular vulnerability. Chamberlin and colleagues examined whether poor olfaction was associated with adverse cerebrovascular events and future stroke risk in the Atherosclerosis Risk in Communities Study. They found that poor olfaction assessed by a single olfaction test was associated with a higher risk of stroke over the following decade [74]. This does not mean that smell loss directly causes stroke, but it suggests that olfactory dysfunction may serve as a marker of vascular, neurodegenerative, inflammatory, or aging-related vulnerability.

This association also overlaps with broader work on frailty. Pleasants and colleagues reported that olfactory dysfunction was associated with frailty risk in older adults, with olfactory function assessed using the 12-item Sniffin’ Sticks Test and frailty measured using both the Fried Frailty Phenotype and the Cumulative Frailty Index [75]. Older adults with better olfactory function showed lower frailty risk than those with poor olfaction [75]. These findings suggest that olfactory dysfunction may represent more than an isolated sensory problem. It may also reflect systemic vulnerability relevant to aging, vascular health, and recovery potential.

Olfactory dysfunction, particularly impaired odor identification, is also associated with cognitive decline in aging and neurodegenerative disease. A recent meta-analysis found substantial olfactory impairment in mild cognitive impairment and Alzheimer’s disease, with odor identification among the most affected domains [76]. This creates an important attribution problem in older stroke populations: an abnormal odor-identification score after stroke may reflect the index lesion, pre-existing neurodegeneration, vascular cognitive impairment, or a mixed vascular-neurodegenerative process rather than a stroke-specific sensory deficit alone. The same interpretative caution applies to synucleinopathies, in which olfactory dysfunction can precede or accompany overt cognitive and motor symptoms.

Limited vascular-dementia and cerebral-small-vessel-disease evidence points in the same direction. In a clinic-based comparison, patients with vascular dementia and patients with Alzheimer-type dementia both showed impaired odor identification relative to controls, and olfactory testing did not reliably distinguish between the two dementia syndromes [77]. More recently, a study of 193 patients with cerebral small vessel disease and 164 controls found poorer odor identification in the small-vessel-disease group; olfactory performance was associated with structural/functional network abnormalities and with subsequent cognitive decline over 24 months [78]. These observations are consistent with a contribution from white-matter disease and network disconnection, but they do not establish that olfactory impairment is specific to vascular pathology.

Clinically and in research, interpretation should therefore incorporate premorbid smell history when available, collateral history from family or caregivers, baseline and longitudinal cognitive testing, and MRI markers of chronic vascular burden such as white-matter hyperintensities, lacunes, and other small-vessel-disease features. A new olfactory deficit temporally linked to a focal stroke is more supportive of a cerebrovascular contribution, whereas longstanding smell loss, progressive cognitive change, or extensive pre-existing small-vessel disease raises the likelihood of mixed causation. Longitudinal studies that obtain both olfactory and cognitive measurements before or soon after stroke and repeat them during recovery are needed to separate acute lesion effects from aging-related, vascular, and neurodegenerative trajectories.

## 14. Research Gaps and Future Directions

Although olfactory dysfunction is increasingly recognized as a clinically meaningful consequence of stroke, the literature remains comparatively underdeveloped relative to other post-stroke sensory, motor, cognitive, and swallowing outcomes. Existing studies suggest that smell and taste deficits may be common after stroke, may persist into the chronic phase, and may affect appetite, food liking, safety, and quality of life [19,47,48]. However, mechanisms remain insufficiently defined, and the field still lacks a clear lesion-to-phenotype model that explains why some patients primarily show reduced odor sensitivity while others show impaired odor identification, altered pleasantness, reduced flavor perception, or impaired odor memory.

A major limitation of the current literature is the heterogeneity of stroke populations and the difficulty of causal attribution. Studies often include patients with diverse lesion locations, stroke types, severities, chronicity, cognitive-language deficits, and comorbidities. Potential non-stroke contributors include sinonasal disease, medication effects, smoking, age-related decline, neurodegenerative disease, and viral infection [19,44]. In studies conducted from 2020 onward, prior SARS-CoV-2 infection deserves explicit ascertainment because persistent post-COVID-19 olfactory dysfunction may overlap temporally with stroke-related symptoms [18,22]. Future protocols should record COVID-19 history and timing, premorbid olfactory symptoms, cognitive status, and chronic small-vessel-disease burden so that direct lesion effects can be distinguished from pre-existing or concurrent causes.

**Figure 4:**
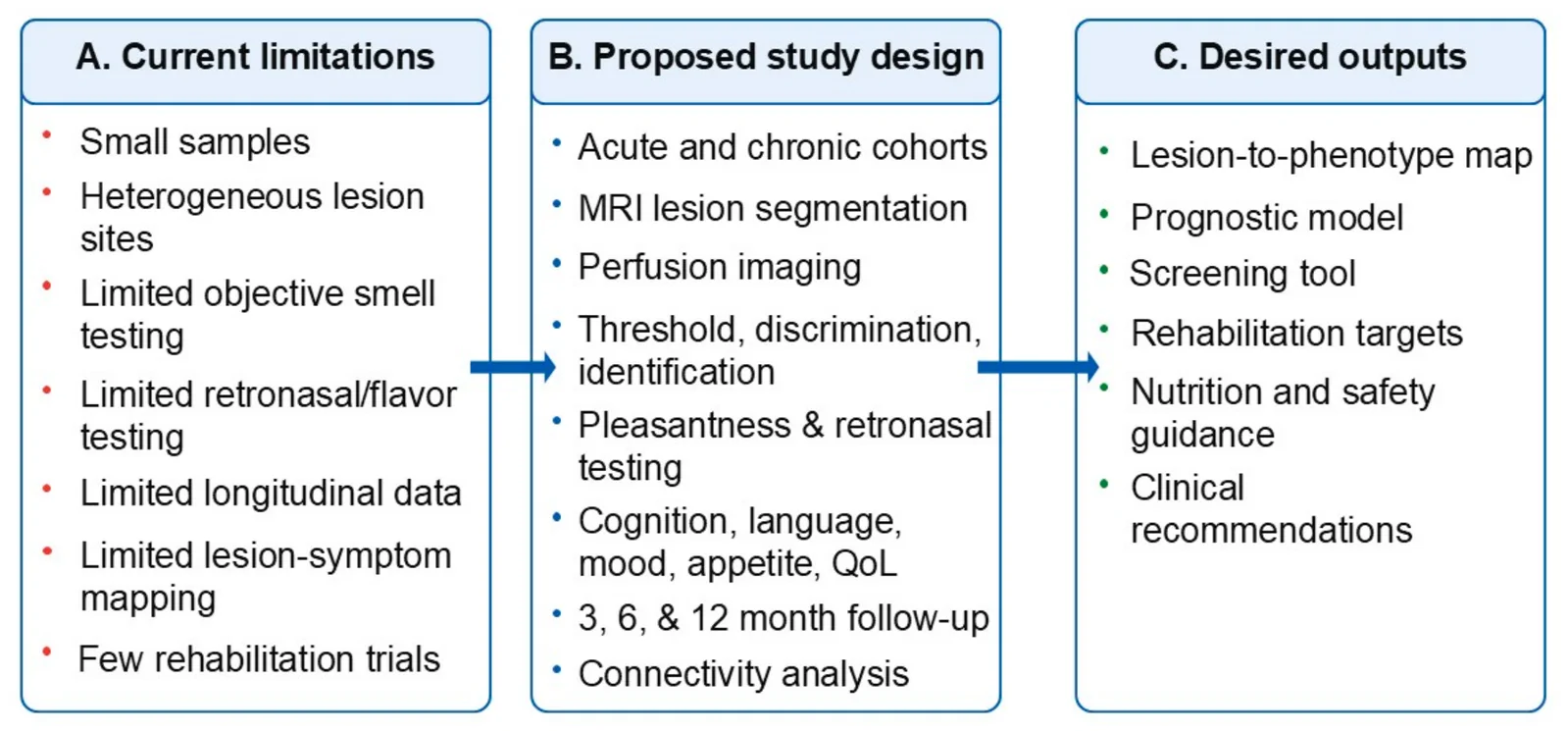
Research gaps and proposed framework for future studies of stroke and olfaction. Current studies of post-stroke olfactory dysfunction are limited by heterogeneous stroke populations, small sample sizes, underuse of objective olfactory testing, limited assessment of retronasal olfaction and flavor, incomplete lesion-symptom mapping, and sparse longitudinal or interventional data. Future studies should combine acute and chronic stroke cohorts with structural MRI, perfusion imaging, lesion segmentation, olfactory threshold, discrimination, identification, pleasantness, retronasal flavor testing, cognitive and language assessment, mood and appetite measures, quality-of-life instruments, and longitudinal follow-up. This approach could identify lesion-specific olfactory phenotypes, clarify mechanisms of recovery or persistence, and guide screening, counseling, and rehabilitation strategies. MRI—magnetic resonance imaging; QoL—quality of life; lesion-symptom mapping—linking lesion sites to behavioral deficits; connectivity analysis—structural or functional network analysis.

Future studies should use a multidimensional but feasible testing framework (Figure 4), separating odor threshold, discrimination, identification, pleasantness, retronasal olfaction, flavor-related outcomes, and odor memory where clinically appropriate. Standardized tools such as the University of Pennsylvania Smell Identification Test and Sniffin’ Sticks can provide validated psychophysical measures [67,68,69], but interpretation should explicitly account for language, memory, attention, neglect, and the accommodations described above rather than treating every low identification score as a pure sensory deficit.

An ideal future study design would enroll patients in the acute or subacute period after ischemic or hemorrhagic stroke and follow them longitudinally into the chronic phase. Participants should undergo structural MRI with lesion segmentation, vascular imaging, perfusion imaging when available, and standardized olfactory and gustatory testing. The olfactory battery should separate peripheral-like sensitivity deficits from central deficits in identification, discrimination, hedonic evaluation, retronasal flavor perception, and odor memory. Taste testing, appetite measures, food liking, nutritional status, mood, cognition, language, and quality-of-life instruments should be included because these domains may mediate or modify the clinical impact of olfactory dysfunction. The 2025 case–control study by Graham and colleagues is a useful model because it assessed smell, taste, hedonic groupings, and food liking in patients with stroke, but future work should add more detailed imaging and longitudinal follow-up to clarify lesion-specific mechanisms [38,47].

Lesion-symptom mapping and network-based analyses are especially important next steps. Voxel-based lesion-symptom mapping could test whether damage to specific regions, such as OFC, insula, piriform cortex, amygdala, entorhinal cortex, hippocampus, or mediodorsal thalamus, predicts specific olfactory phenotypes. Lesion network mapping could then examine whether different lesion locations produce similar olfactory symptoms by disrupting a shared olfactory network rather than a single anatomical site. These approaches are already used in stroke and lesion neuroscience to connect focal lesions with behavioral deficits and distributed brain circuits, and they could help explain cases where olfactory dysfunction occurs despite no visible infarct in the olfactory bulb or primary olfactory cortex [64,65,66].

An additional, exploratory research direction is the oral microbiota. Oral dysbiosis and periodontal disease have been linked in the broader literature to systemic inflammation, endothelial dysfunction, atherosclerotic risk, and stroke-related vascular pathways [79]. A 2026 cross-sectional study of 108 participants reported distinct salivary microbial community patterns in patients with post-stroke cognitive impairment compared with post-stroke patients with normal cognition and healthy controls; several taxa correlated with MMSE and MoCA scores, but the authors appropriately characterized the findings as hypothesis-generating and in need of external validation [80]. No direct evidence currently links oral microbiota composition to post-stroke olfactory dysfunction. Nevertheless, because inflammation, blood–brain barrier dysfunction, cerebral small vessel disease, and neurodegeneration are plausible shared pathways, future longitudinal studies could test whether oral health or microbial signatures modify post-stroke olfactory and cognitive outcomes. This possibility should be regarded as a research hypothesis, not a confirmed mechanism.

Another major gap is the lack of interventional and rehabilitation research. At present, post-stroke olfactory dysfunction is rarely screened systematically, and there is little stroke-specific evidence on whether olfactory training, nutritional counseling, flavor enhancement, safety education, or targeted cognitive-sensory rehabilitation improves outcomes [71,73]. Future trials should test whether structured olfactory training or combined smell-taste-flavor rehabilitation can improve odor identification, food enjoyment, appetite, nutritional status, safety awareness, and quality of life.

Overall, the field needs a shift from descriptive studies toward lesion-informed, longitudinal, multimodal research (Figure 4). The most valuable future studies would combine standardized smell and taste testing, detailed lesion mapping, perfusion imaging, cognitive and affective assessment, appetite and nutrition measures, quality-of-life outcomes, and follow-up across recovery stages. Such work could produce a clinically useful lesion-to-phenotype map, identify predictors of recovery or persistence, clarify whether olfactory impairment contributes to post-stroke nutritional risk, and establish evidence-based screening and rehabilitation recommendations.

## 15. Conclusions

Olfactory dysfunction after stroke is likely underrecognized, undermeasured, and undertreated. The olfactory system is a distributed network rather than a simple linear pathway, and stroke can disrupt smell through direct injury to olfactory structures, impairment of higher-order cortical and limbic regions, thalamic involvement, diaschisis, hypoperfusion, secondary degeneration, altered sniffing, and cognitive or affective mechanisms. As a result, post-stroke olfactory dysfunction may present not only as anosmia or hyposmia, but also as impaired odor identification, altered pleasantness, reduced flavor perception, appetite disturbance, impaired odor memory, and reduced odor-guided behavior.

Because self-report is unreliable, objective and multidimensional assessment is necessary. Clinically, olfactory dysfunction matters because it affects nutrition, safety, quality of life, mood, social behavior, and rehabilitation engagement. Although stroke-specific treatment evidence remains limited, screening, safety counseling, evaluation of reversible contributors, nutrition support, and consideration of olfactory training are reasonable current steps.

Future research should prioritize standardized olfactory phenotyping, acute-to-chronic longitudinal cohorts, lesion-symptom mapping, network-based biomarkers, perfusion imaging, and rehabilitation trials with meaningful patient-centered outcomes. Recognizing olfactory dysfunction as part of the post-stroke syndrome may improve patient safety, quality of life, and recovery while opening an important and currently underdeveloped area of stroke neuroscience.

## Figures and Tables

**Table 1:** Selected human studies of olfactory dysfunction after stroke. The studies differ substantially in design, timing, outcome measures, and sample size; percentages should therefore not be interpreted as directly comparable prevalence estimates.

Study	Design/Sample	Assessment	Main Finding	Key Limitation
Cecchini et al. [49]	Pilot study; 3 ischemic stroke patients	Sniffin’ Sticks and olfactory event-related potentials	Psychophysical performance was normal or mildly reduced, while neurophysiological responses could be absent or delayed on the affected side.	Very small, cooperative sample; not a prevalence study.
Wehling et al. [19]	Prospective chronic cohort; n = 78; assessed ~1 year after stroke admission	Odor detection/identification with standardized psychophysical testing plus self-report	A total of 28.2% had hyposmia and 15.4% had functional or complete olfactory loss; self-report did not reliably identify objective impairment.	Single chronic cohort; age and stroke severity influenced performance.
Okamoto et al. [44]	Acute ischemic stroke; 19 with thalamic hypoperfusion and 9 stroke controls	Odor detection, recognition, identification; SPECT perfusion imaging	Thalamic hypoperfusion group had worse odor identification and recognition thresholds despite similar absolute detection thresholds.	Small mechanistic cohort; selected on perfusion pattern.
Kültür et al. [50]	MRI case–control study; 41 stroke patients and 41 controls	MRI measures of olfactory bulb/sulcus and central smell-related regions	Stroke group showed smaller peripheral and central smell-related structures; left olfactory bulb volume decreased with longer stroke duration.	Imaging study; does not provide a psychophysical prevalence estimate.
Schön et al. [48]	Cross-sectional; n = 128 (57 acute, 71 chronic)	Structured interview/adapted Taste and Smell Survey	A total of 12% reported smell dysfunction and 38% reported taste dysfunction.	Subjective assessment; requires validation with objective smell/taste testing.
Graham et al. [47]	Age/sex-matched case–control; 47 stroke patients and 141 controls	Smell and taste performance, hedonic measures, food liking	Stroke group had poorer smell/taste performance; 50% were classified as anosmic and 46.5% had reduced taste ability.	Clinic sample; cross-sectional and not designed to estimate population prevalence.

## Data Availability

No new data were created or analyzed in this study. Data sharing is not applicable to this article.
